# Outcomes of partial vs. complete repair for large to massive rotator cuff tears: a systematic review and meta-analysis

**DOI:** 10.1016/j.xrrt.2026.100834

**Published:** 2026-07-27

**Authors:** Sadiq Alaqaili, Yahya Ali, Tarek Benzouak, Adel Altarkait, Khaled Alrajhi, Sarav Shah, Jason Corban

**Affiliations:** a Department of Orthopedic Surgery, McGill University Health Centre, Montreal, Quebec, Canada; b King's College London, London, UK; c Department of Orthopaedic Surgery, Jaber Al-Ahmad Hospital, Kuwait City, Kuwait; d Department of Orthopaedic Surgery, New England Baptist Hospital, Boston, MA, USA

**Keywords:** Massive rotator cuff tears, Partial rotator cuff tears, Complete arthroscopic repair, Partial, Arthroscopic repair, Irreparable rotator cuff tear, Functional outcomes, Meta-analysis

## Abstract

**Background:**

Large to massive rotator cuff tears are challenging to repair, with partial and complete techniques showing variable outcomes. The purpose of this study is to systematically compare clinical, functional, and structural outcomes following partial and complete repairs.

**Methods:**

This Preferred Reporting Items for Systematic Reviews and Meta-Analyses‑compliant systematic review screened MEDLINE, Embase, and Cochrane Central Register of Controlled Trials through June 15, 2025. Study quality was assessed using Risk Of Bias In Non-randomized Studies of Interventions. Included studies involved patients with large (≥ 3 cm) or massive (≥ 5 cm, ≥ 2 tendons, or ≥ 30 cm^2^) rotator cuff tears, comparing arthroscopic partial vs. complete repair and reporting at least one clinical or functional outcome. Only comparative human studies published in English were considered. Primary outcomes were post-operative range of motion and strength; secondary outcomes included functional scores, acromiohumeral distance, structural failure rates, post-operative tear size, and post-operative pain score. Data were pooled using random-effects meta-analysis with standardized mean differences and odds ratios with 95% confidence intervals.

**Results:**

Nine studies with 508 patients (220 partial and 288 complete repairs) met inclusion criteria. Complete repair demonstrated greater post-operative strength recovery (standardized mean difference = −0.363, 95% confidence interval: [−0.685, 0.042], *P* = .027; I^2^ = 0%). Forward flexion and internal rotation were comparable between groups with negligible heterogeneity (I^2^ = 0% for both), whereas external rotation showed no difference but substantial heterogeneity (I^2^ = 73.0%). Patient-reported outcomes were largely similar; American Shoulder and Elbow Surgeons and Subjective Shoulder Value did not differ (I^2^ = 0% for both), while Constant scores favored complete repair but with substantial heterogeneity (I^2^ = 67.2%). Across studies that performed post-operative imaging, rates of imaging-defined structural failure/progression were similar, although imaging protocols and failure definitions varied.

**Conclusion:**

In comparative studies of large-to-massive rotator cuff tears, partial repair achieved clinical improvement comparable to complete repair across most patient-reported and range-of-motion outcomes. Complete repair showed modest but consistently superior outcomes in post-operative strength recovery, whereas differences in composite functional scores were less consistent and sensitive to between-study heterogeneity and tear-severity mix. Interpretation of these findings is further limited by tear characteristics and reparability which heavily influenced the repair strategy implemented. Accordingly, these findings should not be interpreted as evidence of equivalence between repair techniques in otherwise repairable tears.

Large to massive rotator cuff tears are typically defined as full-thickness defects involving 2 or more tendons or measuring more than 5 cm in size.^29^ They account for approximately 40% of all rotator cuff tears and have been classified using various systems based on tear size, tendon involvement, and tear pattern.^8^^,^^29^ These injuries are often accompanied by tendon retraction, poor tissue quality, and advanced fatty infiltration of the rotator cuff muscles, all of which complicate surgical repair and reduce the likelihood of successful anatomic healing.^1^^,^^8^^,^^12^ Chronic degeneration, fixed retraction, and poor biological environment frequently limit reparability, making the classification clinically relevant for surgical decision-making.^11^ The primary aim of surgical rotator cuff repair is to relieve pain and restore function by re-establishing balanced force couples in the shoulder.^31^ Complete tendon repair, when feasible, is commonly regarded as the preferred surgical approach, aiming to restore the native tendon footprint and biomechanical integrity. However, in cases where full restoration is not technically possible due to irreparable tissue loss or excessive tension, partial repair techniques are often employed to optimize shoulder kinematics and improve function without complete anatomic reconstruction.^8^ Evidence from several studies indicates that both complete and partial repair techniques for large or massive rotator cuff tears have inherent limitations.^2^^,^^9^^,^^10^^,^^15^^,^^17^, ^18^, ^19^^,^^21^^,^^24^ Complete repair is often associated with a high structural failure rate—frequently exceeding 40%—despite the goal of anatomic restoration. Partial repair, while generally more technically achievable, may yield lower tendon healing rates, although short-term functional outcomes can be comparable to complete repair. This trade-off raises important questions about which strategy offers superior long-term benefits in terms of pain relief, functional recovery, and structural integrity. There is currently no clear consensus on whether complete or partial repair yields better outcomes in this challenging patient subgroup. Previous studies have been limited by small sample sizes, heterogeneous populations, variable surgical techniques, and inconsistent definitions of reparability.^2^^,^^9^^,^^10^^,^^15^^,^^17^, ^18^, ^19^^,^^21^^,^^24^ To the best of our knowledge, no meta-analysis has yet addressed these limitations by synthesizing the available evidence comparing arthroscopic partial vs. complete repair specifically in large to massive rotator cuff tears.

To address this uncertainty, we conducted this systematic review and meta-analysis to compare arthroscopic partial and complete repair in patients with large to massive rotator cuff tears. Our primary outcomes were post-operative range of motion (ROM) and strength, while secondary outcomes included functional scores, structural integrity, and structural failure rates. We hypothesize that complete repairs would be associated with superior structural and strength outcomes; however, we expect comparable functional outcomes between both surgical techniques.

## Methods

### Study protocol and registration

This research was conducted in accordance with the Preferred Reporting Items for Systematic Reviews and Meta-Analyses guidelines.^25^ The study protocol was retrospectively recorded in the International Prospective Register of Systematic Reviews with the registration number: 1080494. Ethical approval was not required, as this study involved only previously published literature.

### Eligibility criteria

Studies were eligible for inclusion if they enrolled patients with large rotator cuff tears, defined as those measuring at least 3 cm, or massive tears, defined as those measuring at least 5 cm, involving 2 or more tendons, or with a tear area of 30 cm^2^ or greater. The intervention of interest was arthroscopic partial repair, compared directly with arthroscopic complete repair.

Eligible studies were required to report at least one relevant clinical or functional outcome, including ROM, strength, functional scores, structural integrity, or structural failure rates. Only comparative human studies published in English were considered. Exclusion criteria included animal or cadaveric studies, abstracts, case reports, commentaries, editorials, expert opinions, conference presentations, review articles, and studies not reporting the outcomes of interest.

Given that tear severity and reparability are key determinants of whether a complete footprint coverage repair is feasible (and therefore whether patients are selected for partial repair), we a priori extracted each study's tear-size definition, reported baseline tear characteristics, explicit intraoperative reparability criteria, post-operative imaging protocol (if any), and the study-specific definition of structural failure. These elements were summarized to make the clinical comparability between cohorts transparent.

### Literature search strategy

Our search was conducted across multiple electronic databases, including MEDLINE, Embase, and the Cochrane Central Register of Controlled Trials, with the final search performed on June 15, 2025. All relevant articles were retrieved from database inception.

Reference lists of all included studies were also reviewed to identify additional relevant publications not captured in the initial search. Duplicate records were removed, and all eligible studies were rigorously evaluated by the research team for final inclusion. To capture unpublished or ongoing studies, additional searches were carried out in the World Health Organization International Clinical Trials Registry and the International Standard Randomized Controlled Trial Number Register. No language restrictions were imposed during the search. The detailed search strategy included the following: (("rotator cuff tear" OR "massive rotator cuff tear" OR "mRCT" OR "large rotator cuff tear") AND ("partial repair" OR "partial arthroscopic repair" OR "arthroscopic partial repair" OR "complete repair" OR "full repair" OR "anatomic repair" OR "complete arthroscopic repair")).

### Selection of studies

Two authors independently screened the titles and abstracts of all identified studies (Y.A. and A.A.).

Full-text versions of relevant studies were obtained, and those meeting the eligibility criteria were selected for inclusion. Any discrepancies in study selection were resolved through group consensus.

### Data extraction and outcomes

Data were extracted using a standardized collection form which was pilot-tested on a random selection of articles and refined based on feedback. The extraction sheet captured: (1) study characteristics (first author, year of publication, corresponding author's country, journal, study design, sample size, participants' clinical condition, type of intervention, and comparator); (2) baseline demographics (age, gender, pre-operative tear size and acromiohumeral distance (AHD), involvement of the dominant arm, and fatty infiltration); and (3) primary and secondary outcomes.

The primary outcomes included ROM in forward flexion, external rotation, internal rotation, and strength. The secondary outcomes included functional scores such as the University of California, Los Angeles (UCLA) Shoulder Score, Constant Murley score, Subjective Shoulder Value (SSV), American Shoulder and Elbow Surgeons (ASES) score, and visual analog scale. Post-operative measures such as tear size, AHD, and structural failure rate were also included.

Three authors independently performed data extraction, with any discrepancies resolved through discussion. The distinction between primary and secondary outcomes was prespecified to prioritize clinically most relevant endpoints and to structure the analysis, not to influence study eligibility. Article selection was based on study design, population, and comparison of partial vs. complete repair; studies were included if they reported at least one relevant outcome, whether primary or secondary.

### Quality assessment

Risk of bias was evaluated using the Risk Of Bias In Non-randomized Studies of Interventions tool as all included studies were non-randomized.^30^ Two independent authors conducted the quality assessment. Disagreements were resolved through consensus after discussing the discrepancies.

### Data analysis

All meta-analyses were carried out with Comprehensive Meta-Analysis software (version 4).

Anticipating clinical and methodological variability across studies, every outcome was synthesized with a random-effects DerSimonian-Laird model.^7^ Continuous endpoints were expressed as standardized mean differences (SMDs) with 95% confidence intervals (CIs), where positive values indicated superiority of partial repair. The dichotomous endpoint of structural integrity (structural failure) was pooled as odds ratios with 95% CI.

For structural endpoints, we extracted whether post-operative imaging was performed and the imaging-based definition used by each study. Because partial repair intentionally leaves a residual defect, the term “structural failure” was interpreted within each study's framework and, where applicable, conceptualized as progression/enlargement of the residual defect or loss of repair continuity on follow-up imaging rather than the mere presence of a defect.

Functional recovery was evaluated with 4 scoring systems (ie, ASES, Constant, SSV, and UCLA). To ensure like-for-like comparisons, functional outcomes were subgrouped by scoring system, and a separate SMD was calculated for each. Due to studies reporting multiple measurement outcomes within their sample, overall effects were not computed. For outcomes reported at multiple post-operative time points, the measurement closest to the longest follow-up was selected to avoid double-counting and to maximize clinical relevance.

Statistical heterogeneity was examined with Cochran's *Q* test (*P* < .10 denoting heterogeneity) and quantified with the I^2^ statistic, interpreted as low (< 25%), moderate (25%-49%), substantial (50%-74%), or high (≥ 75%). Sensitivity analysis was conducted to assess the influence of individual studies on the overall findings. Studies were sequentially excluded to determine their effect on the significance of the results. All tests were 2-sided, and *P* < .05 was considered statistically significant.

## Results

### Summary of the included studies

**Figure 1:**
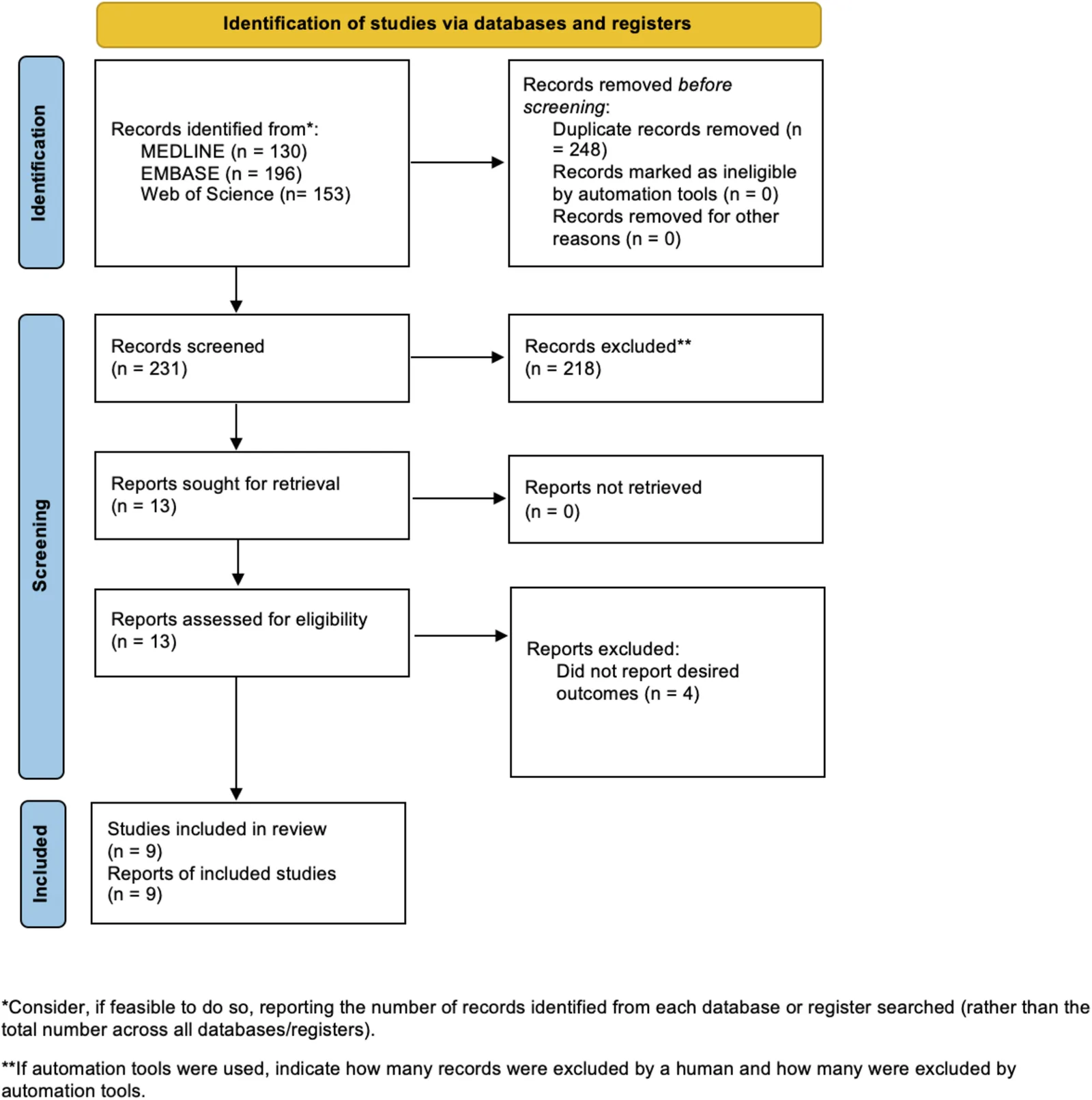
The PRISMA flowchart for literature search and study selection. *PRISMA*, Preferred Reporting Items for Systematic Reviews and Meta-Analyses.

Our initial search yielded a total of 479 papers; after removing duplicates, the number of papers available for screening was 231. After screening, 13 studies were sought for retrieval, and of them, 9 met inlcusion and exclusion criteria^2^^,^^9^^,^^10^^,^^15^^,^^17^, ^18^, ^19^^,^^21^^,^^24^ (Fig. 1). The studies spanned 6 countries, including the United States, South Korea, Austria, France, the Netherlands, and Switzerland. The sample sizes across studies ranged from 27-97 participants. The total number of patients included in this meta-analysis was 508; 220 underwent a partial shoulder repair and 288 underwent a complete shoulder repair. The oldest average age across papers was 65 years in the complete repair group and 68 years in the partial repair group. The youngest average age was 61.5 and 60.2 years in the partial and complete repair group, respectively. Patient baseline characteristics are summarized in Table I.

**Table I:** The baseline characteristics of the included studies

Study ID	Sample size		Age (yrs) ± SD		Sex (M/F)		Pre-operative tear size (mm) ± SD		Pre-operative acromiohumeral distance (mm) ± SD		Dominant arm involved? (yes/no)	
	P	C	P	C	P	C	P	C	P	C	P	C
Moser et al, 2007^24^	11	21	-	-	-	-	-	-	-	-	-	-
Iagulli et al, 2012^17^	52	45	64.5 ± 9.5	63.4 ± 12.2	-	-	-	-	-	-	-	-
Kim et al, 2013^19^	19	22	61.5 ±	60.2 ± 5.85	7/12	8/14	31.8 ± 5.3	30.9 ± 5.4	8.3 ± 0.7	8.2 ± 0.8	14/5	17/5
Holtby et al, 2014^16^	73	49	67 ± 9	64 ± 9	48/25	33/16	-	-	-	-	54/19	38/11
Heuberer et al, 2016^15^	22	23	68.0 ± 8.0	65.0 ± 6.8	14/8	15/8	-	-	-	-	18/4	15/8
Godenèche et al, 2016^10^	23	50	-	-	-	-	-	-	-	-	-	-
Besnard et al, 2020^2^	20	44	-	-	-	-	-	-	-	-	-	-
Jeong et al, 2020^18^	33	25	61.9 ± 7.0	61.4 ± 6.3	13/20	11/14	32.5 ± 4.6	32.2 ± 4.3	8.1 ± 1.0	8.2 ± 0.9	26/7	19/6
Farazdaghi et al, 2021^9^	14	13	-	-	-	-	-	-	-	-	-	-
Lee et al, 2024^21^	26	45	61.6 ± 5.5	62.9 ± 5.8	13/21	22/34	32.3 ± 3.8	31.2 ± 3.7	8.1 ± 1.0	8.5 ± 0.7	26/8	47/9

*SD*, standard deviation; *M*, male; *F*, female; *P*, partial repair group; *C*, complete repair group.

Tear-severity definitions varied across studies. Three studies enrolled mixed large-to-massive tears using an anteroposterior threshold of ≥ 3 cm and/or ≥ 2-tendon involvement, whereas the remaining studies focused on massive tears (typically ≥ 5 cm and/or ≥ 2 tendons and/or ≥ 30 cm^2^).

Across most cohorts, allocation to partial repair reflected intraoperative irreparability (inability to mobilize and cover the footprint without excessive tension) rather than surgeon preference alone.

To make this selection-by-indication transparent, Table II summarizes each study's tear definition, reparability criteria, post-operative imaging protocol, and structural failure framework.

**Table II:** Tear definition, reparability/selection framework, post-operative imaging protocol, and structural failure definition across included studies

Study ID	Definition used	Baseline tear size (mean ± SD)	% classified as massive tears	Tendon involvement	Retraction	Fatty infiltration (Goutallier grade)	Pre-operative acromiohumeral distance (mean ± SD)	Reparability criteria used	Post-operative imaging + timing	Retear/failure definition
Moser, 2007^24^	A massive rotator cuff tear was defined as a tear of at least 5 cm that involved at least 2 tendons (only massive tears in this study)	N/A	100	N/A	N/A	N/A	N/A	N/A	N/A	N/A
Iagulli, 2012^17^	A diameter greater than 5 cm, 9 involving 2 or more tendons, 13 having an area of 30 cm^2^ or greater, and 5 as noted intraoperatively.	Partial: medial to lateral: 6.07 ± 0.26 cm, anterior to posterior: 6.54 ± 0.64 cm, overall area: 39.73 ± 4.57 cm^2^/Complete: medial to lateral: 5.96 ± 0.21 cm, anterior to posterior: 5.91 ± 0.70 cm, overall area: 35.20 ± 4.36 cm^2^	100	N/A	N/A	N/A	N/A	Once the rotator cuff tissue was mobilized to its fullest extent, careful assessment was made as to which repair configuration would yield the most complete repair at the lowest tension possible. Margin convergence 6 was often used to lateralize the free margin of the rotator cuff tear and to minimize strain at the repair site. Suture anchors were placed to repair the rotator cuff to the bone by single-row or double-row fixation techniques depending on tissue quality and mobility. Attempts were made to completely repair all massive rotator cuff tears. However, if reapproximation of the tendon to bone could not be accomplished even after extensive rotator cuff mobilization without undue tension on the mobilized tendon, repair of only the adequately mobilized tissue was carried out. Whether a partial or complete repair became possible after Vol. 40, No. 5, 2012 Partial vs. Complete Repair Massive Tears 1023 rotator cuff mobilization, rotator cuff mobilization attempts usually started with an extensive capsular release along with release of any peritendinous adhesions. If additional rotator cuff mobilization was still thought to be necessary, an anterior or posterior interval slide was accomplished.	MRI no timing mentioned	N/A
Kim, 2013^19^	A rotator cuff tear with an anterior-to-posterior diameter of ‡3 cm or at least a 2-tendon involvement noted in the pre-operative MRA and intraoperative period (no difference in large and massive tears was stated, only said this is the definition for large-to-massive tears)	Complete: 30.9 ± 5.4, Partial: 31.8 ± 5.3	N/A	Complete group: 8 subscapularis, 9 SLAP lesion or bicep tendon involvement Partial group: 7 subscapularis, 9 SLAP and bicep tendon involvement	N/A	Degree of fatty infiltration in the supraspinatus and infraspinatus muscles was categorized as follows: stage 0 indicated a completely normal muscle without any fatty streak; stage 1, occasional fatty streaks in the muscle; stage 2, obvious fatty infiltration but with still more muscle than fat; stage 3, equal amount of fat and muscle; and stage 4, more fat than muscle. Complete: supraspinatus stage 3.3, infraspinatus stage 2.9/partial: supraspinatus stage 3.4 and infraspinatus 2.9	Complete: 8.2 ± 0.8 mm, partial: 8.3 ± 0.7 mm	Complete: 17 simple repair, 5 suture bridge. Partial: 15 simple repair, 4 suture bridge	MRA pre-operative and at 6 mo	N/A
Godenèche, 2016^10^	Tears involving 2 or more tendons (did not include large tears in this study)	N/A	100%	N/A	N/A	Stage I in 28 shoulders (38.4%), stage II in 34 shoulders (46.6%), and stage III in 11 shoulders (15.1%).	N/A	The repairability of each tear was confirmed intraoperatively. In 50 shoulders (31.5%), all torn tendons were repaired (complete), while in 23 shoulders (68.5%), some of the torn tendons were repaired (partial), but at least one tendon could not be repaired due to severe tissue degeneration or retraction.	CT and MRI pre-operatively, USS at least 2 mo post-operatively	N/A
Heuberer, 2016^15^	Tears involving more than 2 tendons and with a diameter width of at least 5 cm (massive tears only)	N/A	100%	Complete group: subscapularis: 2 full-tendon, full thickness/14 partial tendon full thickness/5 partial tendon partial thickness, LHB: 13 partial rupture or inflamed/7 dislocated, supraspinatus: 23 full tendon full thickness, infraspinatus: 11 full-tendon full thickness, 12 partial tendon full thickness, teres minor: partial tendon full thickness; partial: subscapularis: 6 full tendon full thickness, 11 partial tendon full thickness, 5 partial tendon partial thickness, LHB: 11 partial or inflamed, 3 dislocated, supraspinatus: 22 full tendon full thickness, infraspinatus: 13 full tendon full thickness, 9 partial tendon full thickness, teres minor: 1 full tendon full thickness, 1 partial tendon full thickness	N/A	Complete: 1.0 ± 0.6; partial: 1.5 ± 0.5 global fatty index	N/A	Whenever decision of an attempt for partial or complete rotator cuff reconstruction was made, extensive tendon releases (double, anterior, or interval slide in continuity [26]) with resection of the coracohumeral ligament of the coracoid base, juxtaglenoidal as well as extra-articular tendon and muscle mobilization were performed	MRI or USS	Thinning of the tendon without or minor discontinuity on one image was regarded as partly reruptured and obvious discontinuity in more than one slice was regarded as retear.
Besnard, 2020^2^	Three-tendon tears or full-thickness 2-tendon tears (massive tears only)	N/A	100%	N/A	N/A	Stage 1: 25, stage 2: 30, stage 3: 9	N/A	The repairability of each tear was noted as ‘complete’ if all torn tendons were restored to their anatomic footprints, or ‘partial’ if one or more tendons could not be reinserted due to substantial degeneration or retraction	Pre-operative CT or MRI	N/A
Jeong, 2020^18^	The presence of a large and massive rotator cuff tear with an anteroposterior dimension of ‡3 cm or involvement of 2 or more tendons identified during a pre-operative magnetic resonance arthrography (MRA) and arthroscopic evaluation	Complete: 32.2 ± 4.3; partial: 32.5 ± 4.6	N/A	A biceps tendon lesion was identified in 13 patients (52%) in the complete-repair group and 16 patients (48%) in the partial-repair group. These patients underwent biceps tenotomy or tenodesis. A concomitant partial or full-thickness subscapularis tear requiring repair was identified in 9 patients (36%) in the complete-repair group and 11 patients (33%) in the partial-repair group. The supraspinatus and infraspinatus tears were repaired with use of either a single-row (19 patients in thecomplete-repair group and 25 patients in the partial-repair group) or a double-row suture-bridge technique (6 patients in the complete-repair group and 8 patients in the partial-repair group).	N/A	Supraspinatus: Complete pre-operative: 3.2 ± 0.5, post-operative: 3.0 ± 0.5, partial: pre-operative: 3.3 ± 0.6, post-operative: 3.1 ± 0.5/infraspinatus: complete: pre-operative: 2.8 ± 0.6, post-operative: 2.6 ± 0.7; partial: pre-operative: 2.7 ± 0.7, post-operative: 2.7 ± 0.7	Complete: 8.2 ± 0.9 mm; partial: 8.1 ± 1.0	A biceps tendon lesion was identified in 13 patients (52%) in the complete-repair group and 16 patients (48%) in the partial-repair group. These patients underwent biceps tenotomy or tenodesis. A concomitant partial or full-thickness subscapularis tear requiring repair was identified in 9 patients (36%) in the complete-repair group and 11 patients (33%) in the partial-repair group. The supraspinatus and infraspinatus tears were repaired with use of either a single-row (19 patients in the complete-repair group and 25 patients in the partial-repair group) or a double-row suture-bridge technique (6 patients in the complete-repair group and 8 patients in the partial-repair group).	MRA at 6 mo	N/A
Farazdaghi, 2021^9^	Tears larger than 5 cm that involve at least 2 of the rotator cuff tendons (only massive tears)	35.5 ± 17.7, results merged	100%	Tendons involvement; Two 2/27; Three 12/27; Four 13/27	N/A	N/A	N/A	A preliminary decision for arthroscopic repair was based on the size of the tear, the morphology of the involved tendons, and the chronicity of the tear. The final decision of full vs. partial coverage repair was made under direct arthroscopic evaluation of the tear and its ability to mobilize that would indicate adequate capacity for repair. Our definition for partial coverage was solely based on the footprint coverage achieved. Therefore, partial coverage repair was referred to those tears with technical inability to fully cover the footprint.	MRI at 6 mo	• Persistent symptoms such as pain, weakness, stiffness, or pseudoparalysis; • Inability to achieve preinjury activity level; • Prolonged recovery process lasting more than 6 mo
Lee, 2024^21^	A tear with a diameter of at least 3 cm from anterior to posterior or a tear that affected 2 or more tendons, identified on magnetic resonance arthrography (MRA) conducted before the surgical procedure or through arthroscopic examination	Complete: 31.2 ± 3.7 mm; partial: 32.3 ± 3.8 mm	N/A	N/A	N/A	Supraspinatus: complete: pre-operative: 3.1 ± 0.4, post-operative: 3.0 ± 0.3, partial: pre-operative: 3.3 ± 0.4, 3.2 ± 0.4, infraspinatus: complete: pre-operative: 2.6 ± 0.5, post-operative: 2.5 ± 0.5, partial: pre-operative: 2.8 ± 0.5, post-operative: 2.7 ± 0.5	Complete: 8.5 ± 0.7 mm; partial: 8.1 ± 1.0	The patients were assigned to the partial-repair group (group P) if their injury was repaired as much as possible but a residual defect (uncovered humeral head) remained, even after margin convergence, or to the complete-repair group (group C) if complete repair was performed without any residual defect. Patients who underwent an additional posterior interval slide for complete repair were not included.	MRA at 6 mo	Definition of “failure” was established as pain and functional deficit significant enough to require revision surgery

*SD*, standard deviation; *N/A*, not applicable; *MRI*, magnetic resonance imaging; *MRA*, magnetic resonance angiography; *CT*, computed tomography; *SLAP*, superior labrum anterior to posterior; *USS*, ultrasound scan (ultrasonography); *LHB*, long head of the biceps.

### Summary of the study quality assessment

**Figure 2:**
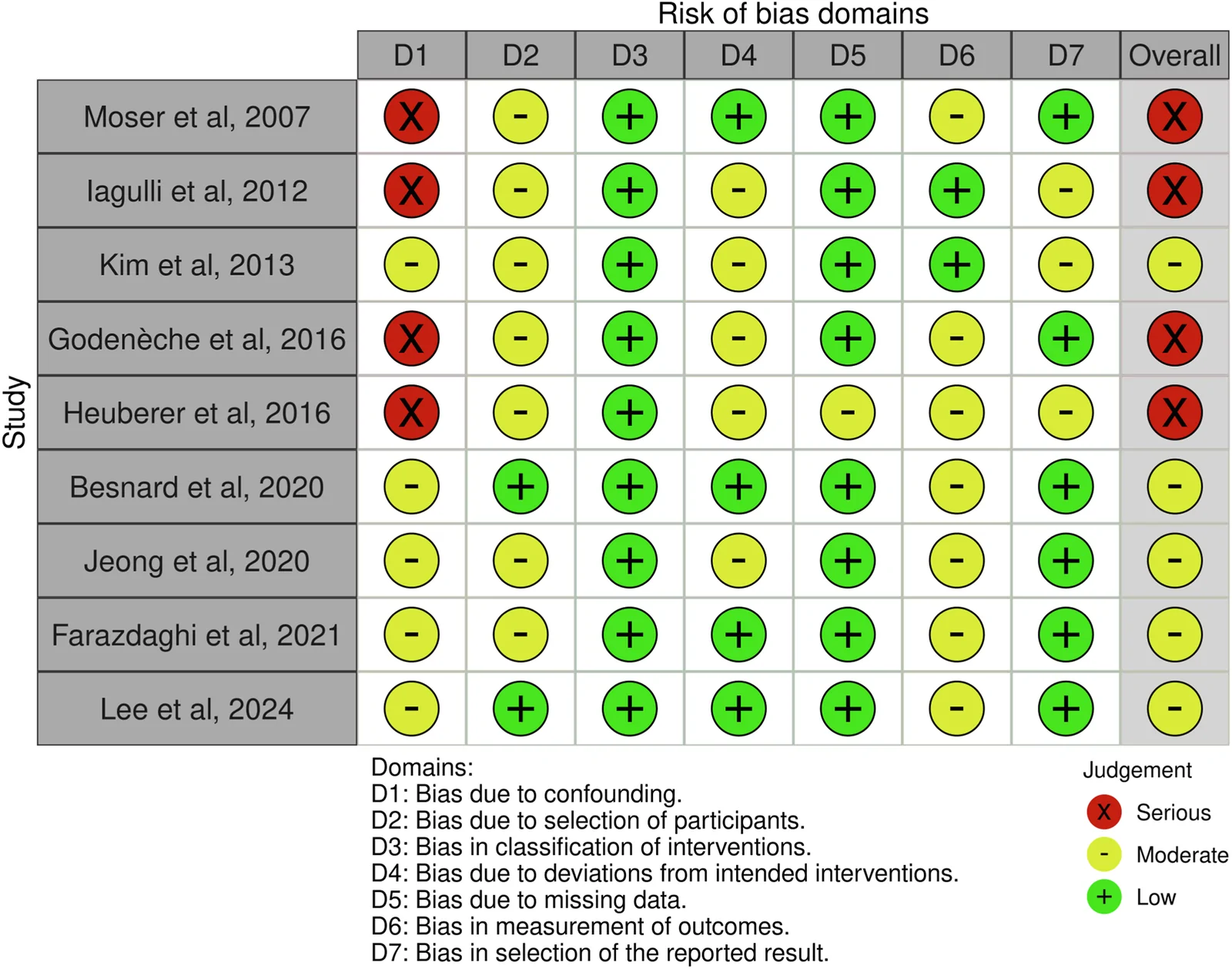
Summary of the non-randomized studies according to the ROBINS-I tool. *ROBINS-I*, Risk Of Bias In Non-randomized Studies of Interventions.

Most papers carried a moderate risk of bias, with 4 of the 9 papers carrying a serious risk due to concerns over confounding factors (Fig. 2).

### Meta-analysis of the primary endpoints

**Figure 3:**
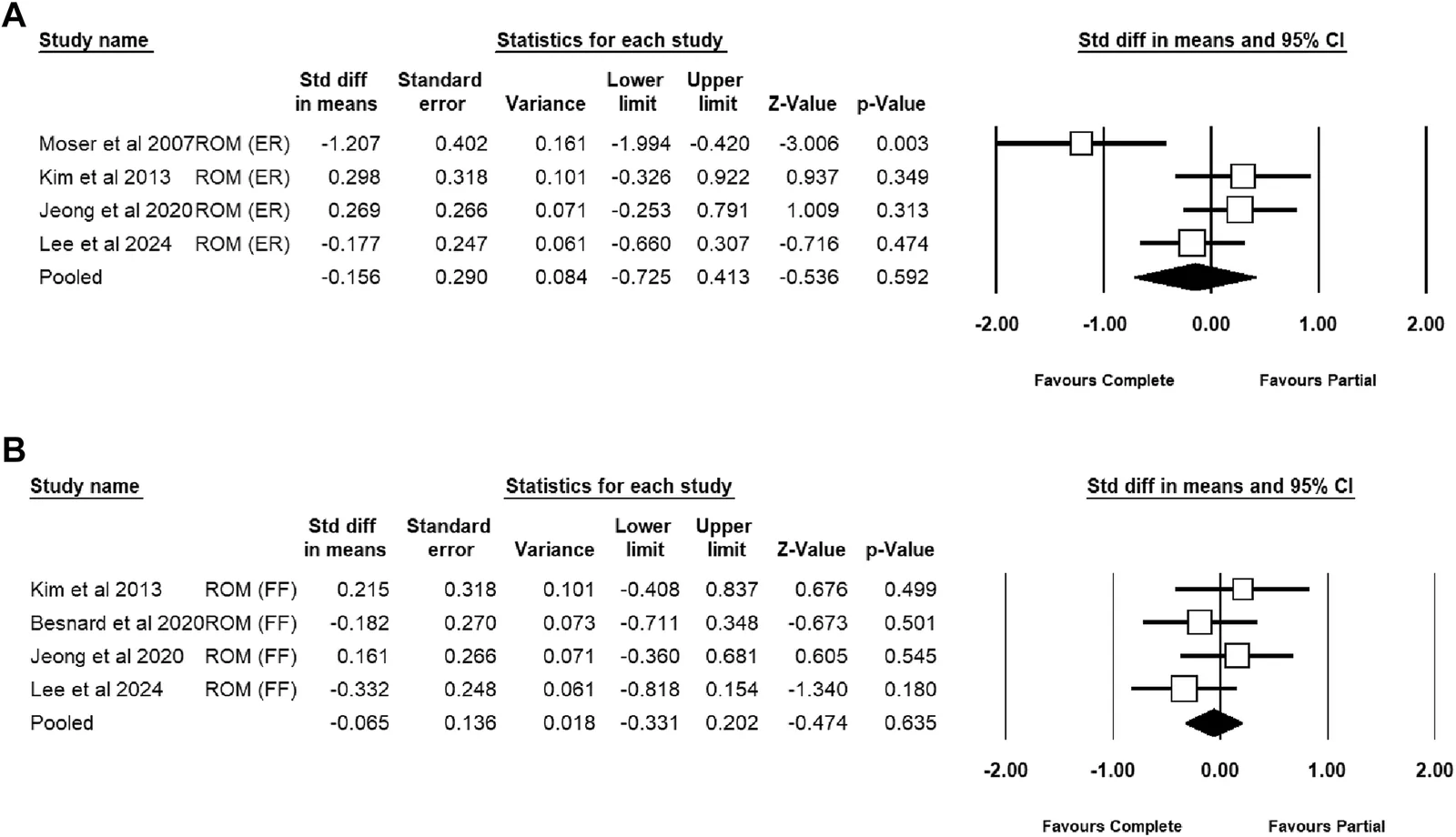

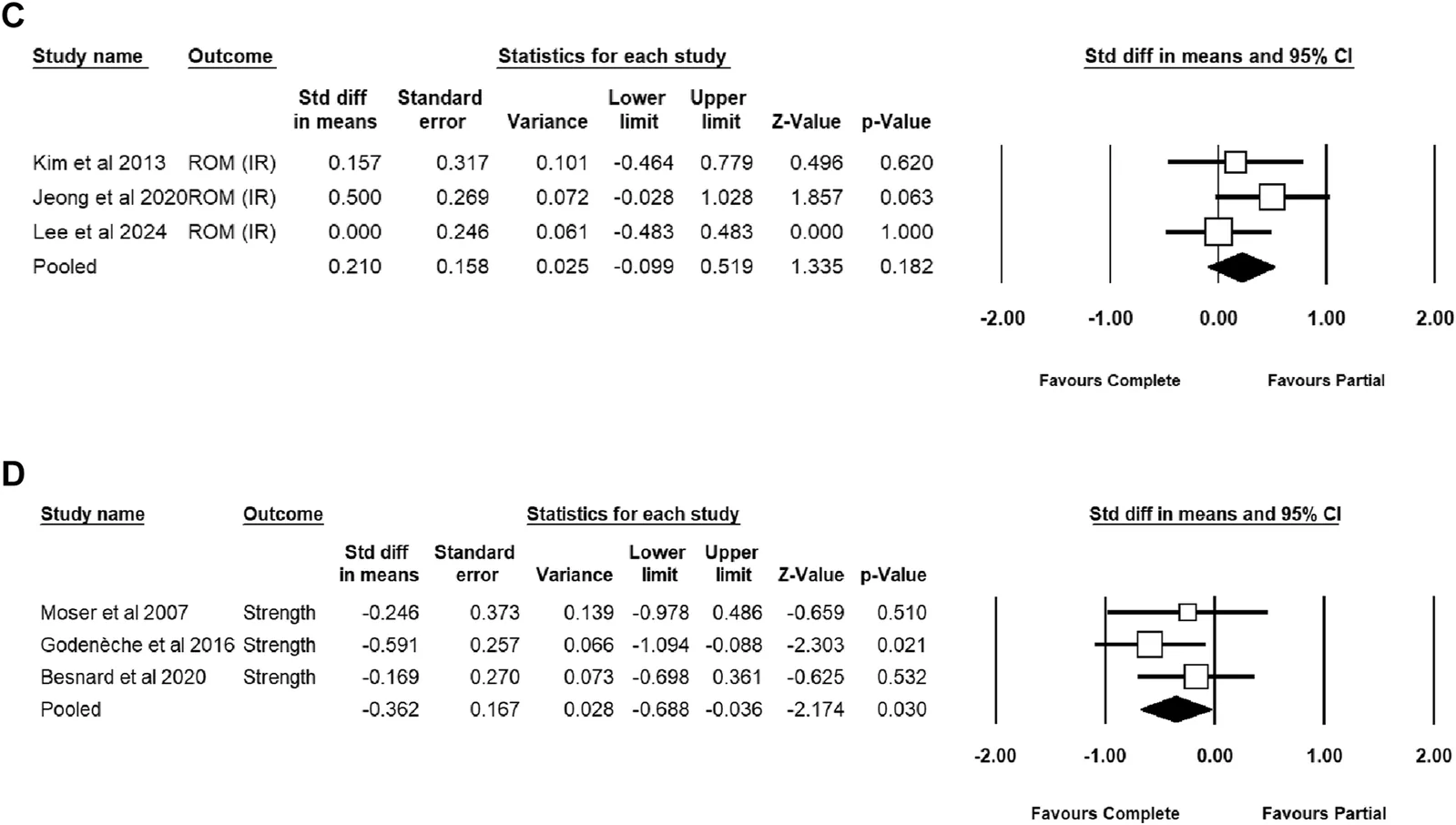
Meta-analysis of the primary endpoints: (**A**) post-operative range of motion for external rotation, (**B**) post-operative range of motion for forward flexion, (**C**) post-operative range of motion for internal rotation, and (**D**) post-operative shoulder-strength recovery.

Across 4 comparative studies encompassing 374 shoulders, there was no statistically significant difference in post-operative external rotation between complete and partial repairs of massive rotator cuff tears. Pooled analysis using a random-effects model indicated clinical equivalence between the 2 techniques (n = 4, SMD = −0.16, 95% CI: −0.73 to 0.41, *P* = .59; Fig. 3*A*), although heterogeneity was substantial (I^2^ = 73%), reflecting considerable between-study variability.

Similarly, analysis of 4 studies revealed no difference in post-operative forward flexion ROM between complete and partial repairs (n = 4, SMD = −0.065, 95% CI: −0.331 to 0.202, *P* = .64; Fig. 3*B*). Consistency across studies was high, with negligible heterogeneity (I^2^ = 0%).

For post-operative internal rotation, 3 comparative studies found no statistically significant advantage for either technique. No statistically significant difference was observed (n = 3, SMD = 0.21, 95% CI: 0.10-0.52, *P* = .18; Fig. 3*C*), and between-study heterogeneity was negligible (I^2^ = 0%).

In contrast, 3 studies reported a small but statistically significant benefit of complete repair over partial repair for post-operative shoulder-strength recovery (n = 3, SMD = −0.36, 95% CI: −0.69 to 0.04, *P* = .03; Fig. 3*D*), with negligible heterogeneity (I^2^ = 0%).

### Meta-analysis of secondary outcomes

A random-effects subgroup analysis of functional scores demonstrated that complete repair was associated with higher Constant scores (SMD = 0.971, 95% CI: 0.093-1.850, *P* = .031); however, heterogeneity was substantial (I^2^ = 67.2%), indicating that this estimate is sensitive to between-study differences (eg, tear-severity mix, rehabilitation protocols, and score administration).

**Figure 4:**
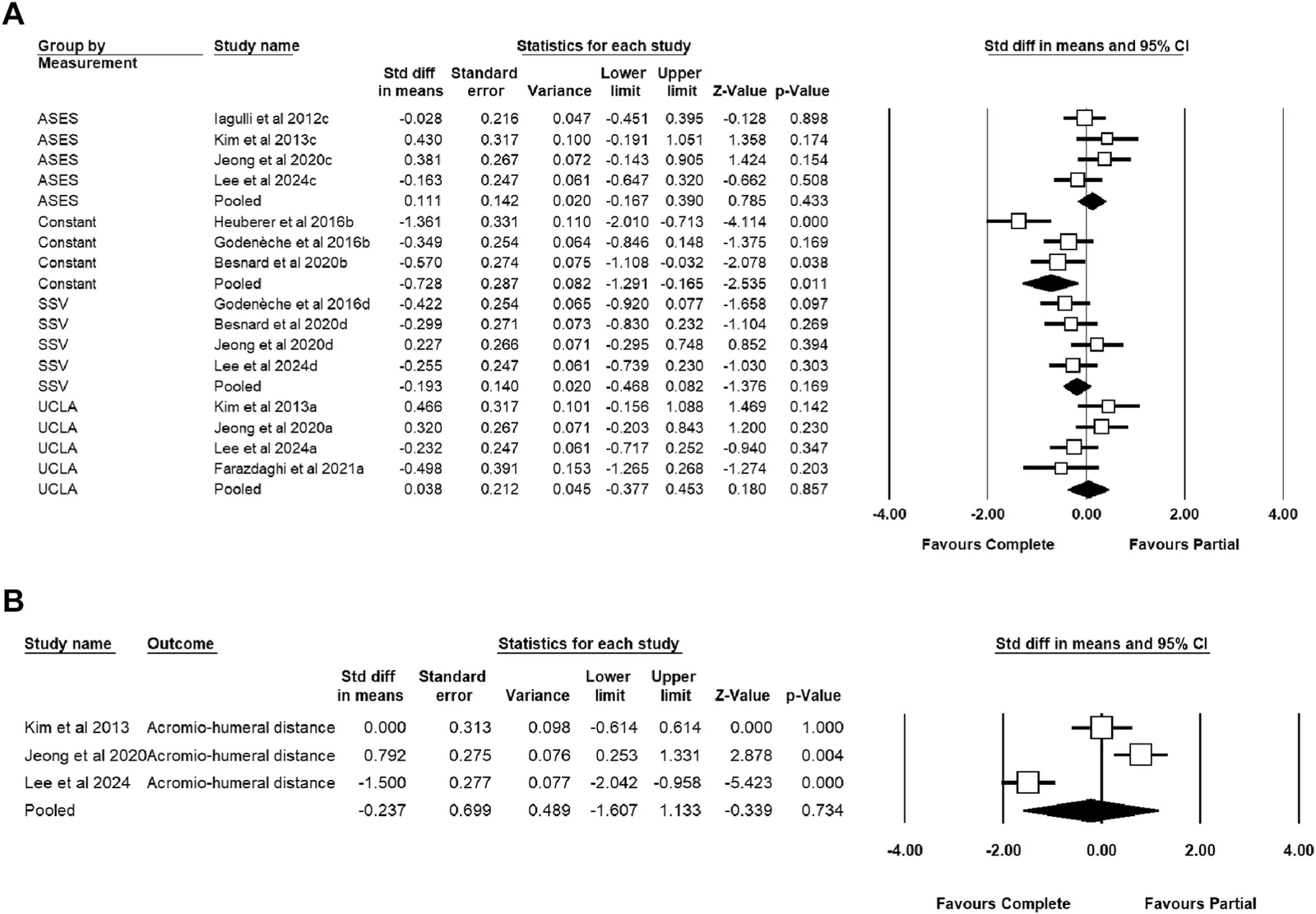

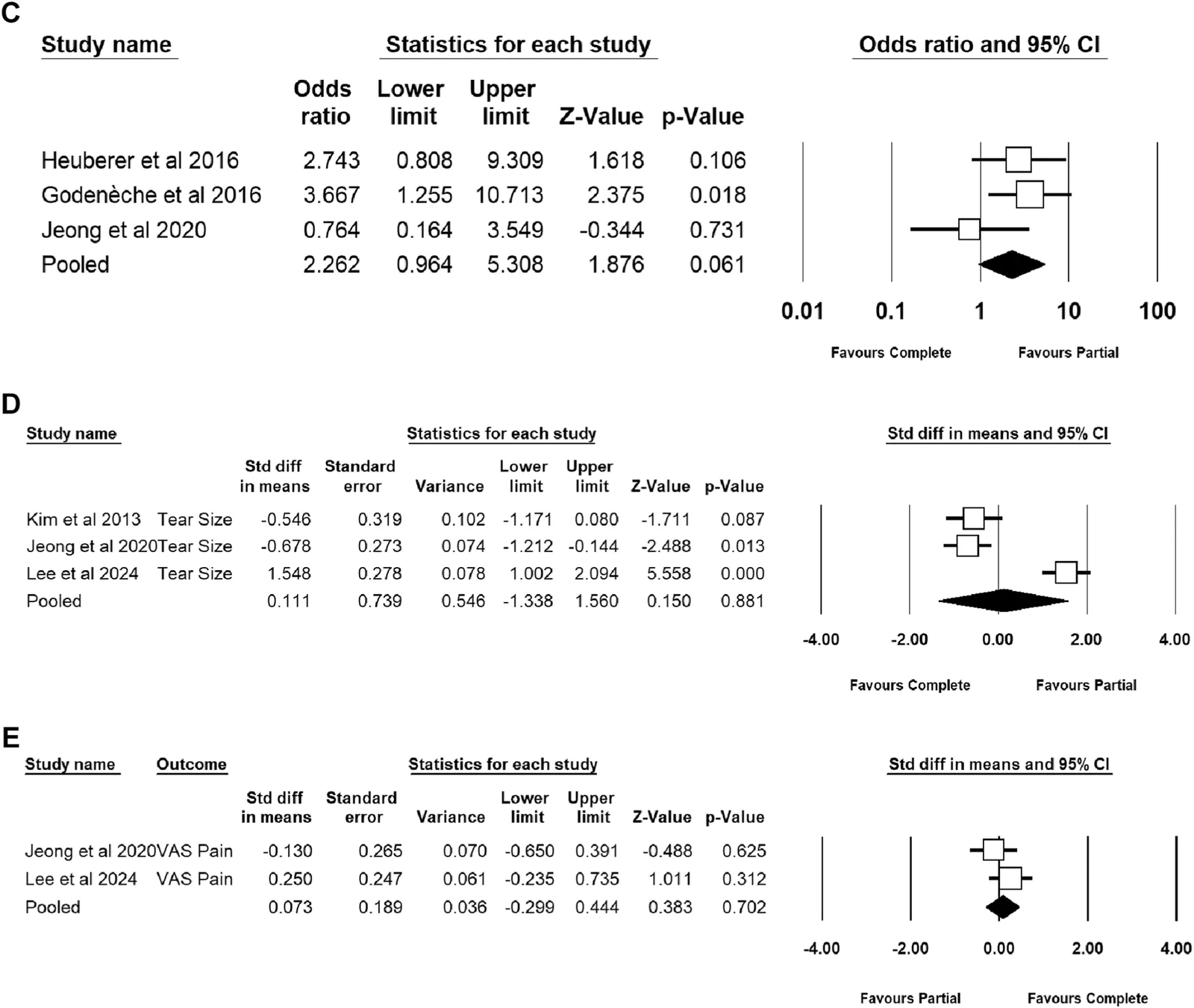
Meta-analysis of the secondary endpoints: (**A**) functional recovery based on 4 scoring systems, (**B**) acromiohumeral distance, (**C**) imaging-defined structural failure/progression, (**D**) post-operative tear size, and (**E**) post-operative pain score.

In contrast, ASES and SSV scores did not differ between groups and showed negligible heterogeneity (ASES: SMD = 0.052, 95% CI: 0.203-0.306, *P* = .692, I^2^ = 0%; SSV: SMD = 0.100, 95% CI: 0.134-0.334, *P* = .401, I^2^ = 0%). UCLA scores were also similar (SMD = 0.038, 95% CI: 0.377-0.453, *P* = .857) with borderline-to-moderate heterogeneity (I^2^ = 50.1%; Fig. 4*A*).

Three studies assessed post-operative AHD. Random-effects synthesis indicated comparable spacing following either technique (n = 3, SMD = −0.237, 95% CI: −1.607 to 1.133, *P* = .734), although heterogeneity was high (I^2^ = 94.4%; Fig. 4*B*).

Structural integrity, evaluated via post-operative imaging, showed no statistically significant difference in structural failure rates between partial and complete repair. All 3 included studies reported similar odds of tendon structural failure (n = 3, odds ratio = 2.262, 95% CI: 0.964-5.308, *P* = .061), with low between-study heterogeneity (I^2^ = 28%; Fig. 4*C*).

Post-operative tear size was reported in 3 studies. Random-effects analysis revealed no significant difference between techniques (n = 3, SMD = 0.111, 95% CI: 1.338-1.560, *P* = .881), indicating comparable structural outcomes, although heterogeneity was high (I^2^ = 94.9%; Fig. 4*D*).

Finally, 2 studies evaluated post-operative pain using a visual analog scale. Pooled analysis demonstrated no significant difference between partial and complete repair (n = 2, SMD = 0.073, 95% CI: 0.299-0.444, *P* = .702), with low heterogeneity (I^2^ = 8.7%; Fig. 4*E*). The sensitivity analysis demonstrated that no single study, including those with a high risk of bias, had a decisive impact on the overall outcomes. These findings were further supported by a funnel plot assessment.

## Discussion

In this systematic review and meta-analysis of 9 comparative studies including 508 patients with large-to-massive rotator cuff tears (220 partial repairs and 288 complete repairs), the principal finding was that partial and complete repairs yielded comparable post-operative ROM and most patient-reported outcome measures (PROMs), while complete repairs were associated with higher constant scores and provided a modest but statistically significant advantage in objective strength recovery. Across included studies, both partial and complete repair groups generally demonstrated post-operative improvements in pain, ROM, and functional outcome measures when pre-operative and post-operative data were reported. The present meta-analysis primarily evaluated comparative post-operative differences between repair strategies rather than within-group change from baseline.

Clinically, our results suggest comparable PROMs between both groups, whereas complete repairs were associated with modestly greater post-operative strength recovery. However, these findings must be interpreted in the context of the non-randomized nature of the available evidence.

Historically, “water-tight repairs” (complete tendon-to-bone restorations) were considered to be the optimal approach to tendon repairs working on the assumptions that greater structural integrity would correlate to superior clinical and functional outcomes. However, early investigations by Harryman et al and Yamaguchi et al challenged this concept by evaluating post-operative repair integrity and demonstrating that structural healing and clinical recovery are not always synonymous.^13^^,^^31^ Many patients maintained adequate clinical function despite tendon defects present on imaging. Similarly, structurally intact repairs did not guarantee superior clinical function. This concept provides an important framework for interpreting the present findings, where comparable patient-reported outcomes may coexist with differences in structural or strength-related measures.

Outcomes with low heterogeneity (I^2^ < 50%)—including strength (I^2^ = 0%), forward flexion (I^2^ = 0%), internal rotation (I^2^ = 0%), ASES (I^2^ = 0%), SSV (I^2^ = 0%), pain (I^2^ = 0%), and imaging-defined structural failure/progression (I^2^ = 27.4%)—support broadly comparable post-operative function between strategies, with a consistent strength advantage for complete repair. By contrast, outcomes with substantial heterogeneity (eg, external rotation ROM, AHD, post-operative tear size, and Constant score) should be interpreted as hypothesis-generating and likely reflect differences in tear-severity mix, measurement methods, and rehabilitation protocols across studies.

When interpreting “functional outcomes,” care must be taken to distinguish subjective PROMs and objective strength. Many commonly reported PROMs particularly the ASES primarily capture pain relief and activities of daily living rather than dynamometric strength.

The original ASES shoulder assessment described by Richards et al is weighted toward pain and activities/function without an independent objective strength domain, whereas the Constant Murley is a 100-point composite outcome measure that allocates 25 points (25%) to objective strength assessment.^5^^,^^26^ Strength is commonly measured with the arm elevated (typically in the scapular plane or approximately 90° of abduction), while resistance or weights are progressively applied, with higher force generation contributing proportionally to the total score. Because strength contributes substantially to the overall Constant score, differences in measured strength can disproportionately influence total score comparisons between treatment groups. Additionally, objective strength assessment within Constant scoring is method-dependent, and variability in testing position and protocol can materially influence measured strength outcomes across studies.^32^ This measurement structure likely explains why our analysis demonstrated a significant advantage for complete repair in Constant score while showing no significant differences for PROMs that are largely pain-driven/ADL-driven.

The clinical relevance of statistically significant between-group differences should also be interpreted in relation to published Minimal Clinically Important Difference (MCID) thresholds. Reported MCID values after rotator cuff repair vary across outcome instruments, patient populations, and methodological approaches, with published estimates for ASES and Constant scores commonly ranging between approximately 6 and 10 points. Accordingly, statistically significant pooled differences should not automatically be interpreted as clinically meaningful when the magnitude of effect is modest.

Pertaining to MCID values and strength, the interpretation of objective strength outcomes warrants additional caution because strength assessment methods were heterogeneous across included studies, including manual testing, dynamometry-based protocols, and composite outcome measures such as the Constant score. Furthermore, direct MCID thresholds for objective strength recovery after rotator cuff repair are less consistently established than for PROMs.

Our findings align with long-term comparative evidence demonstrating similar clinical and PROM outcome despite residual structural differences. Lee et al reported comparable ≥10-year clinical scores and survivorship between partial and complete repair groups despite larger residual defects in the partial repair cohort.^21^ This may be accounted for from numerous factors including (1) aggressive management of the subscapularis to reinforce the suspension bridge concept and (2) biologic “relearning” for deltoid function.^28^ On the other hand, broader evidence indicates that structural integrity and healing are associated with better outcome domains including pain and composite scores, although the magnitude varies by instrument and does not uniformly translate to equivalent recovery across strength related endpoints. In Haque et al’s meta-analysis, intact repairs also showed superior forward elevation strength, underscoring a key interpretive point: PROM ‘equivalence’ does not necessarily imply equivalence in strength.^14^

The absence of a pooled difference in acromiohumeral interval or AHD is biomechanically consistent with the concept that partial repair can restore a stable glenohumeral fulcrum even when the anatomic footprint is not fully reconstructed. Burkhart's “suspension bridge” model supports the premise that restoration of a stable fulcrum permits near-normal elevation kinematics in massive tears.^3^ Mechanistically, this is compatible with restoration of the anterior–posterior force couple and rotator cable–crescent load-sharing, which can maintain humeral head centering despite residual defects.^3^^,^^4^ Concavity compression provides a quantitative framework for this: Lippitt et al demonstrated that increasing compressive load markedly increases resistance to glenohumeral translation, supporting the concept that restored compressive cuff forces can stabilize the humeral head within the glenoid concavity and help resist superior migration.^23^ Although reduced AHD is associated with rotator cuff pathology and superior migration,^27^ pooled findings of the included studies suggest that partial repair can restore sufficient balancing compression and coupling to preserve vertical stability of the glenohumeral joint. Interpretation should remain cautious given substantial heterogeneity in radiographic AHD outcomes in the included studies. Although AHD measurement itself can demonstrate good reliability under standardized conditions, imaging protocols, radiographic positioning, and measurement methodology were not standardized across the included studies.^6^ The included studies did not consistently stratify ROM, strength, or patient-reported outcomes according to post-operative repair integrity, AHD, post-operative tear size, or fatty infiltration. As such, these structural parameters could not be quantitatively correlated with clinical outcomes in the present pooled analysis.

However, the strength advantage noted after complete repair is biologically plausible and consistent with basic muscle mechanics. Maximal force capacity is strongly related to physiological cross-sectional area and effective force transmission through the tendon–bone interface; incomplete restoration of the footprint and tendon bone coupling plausibly limits how much contractile tissue is effectively linked to the humerus, even if humeral head centering is re-established.^22^ This provides a coherent explanation for why kinematics (ROM/AHD) may normalize while maximal kinetics (strength) remain lower, as may be the case with partial repairs. Importantly, some individual cohorts (eg, Jeong et al) have reported no statistically significant between-group differences in strength,^18^ but such null findings are susceptible to limited power and heterogeneity in strength testing methods (manual grading vs. handheld dynamometry vs. isokinetic protocols). By pooling available comparative data, our meta-analysis likely reduces type II error and detects a small but consistent strength advantage for complete repair. Additional comparative studies in this space (including cohorts directly comparing complete repair and partial repair, and in some series débridement) also suggest that complete repair can provide a higher strength ceiling than partial repair, supporting the directionality of our pooled finding.^10^^,^^17^

Reparability and surgical technique are the key factors in clinical decision-making. Tear size and shape, limited tendon mobility or retraction, and tendon quality often constrain feasibility of complete repair in large-to-massive tears.^16^ In such settings, restoring balanced couples and a stable fulcrum via partial repair remains a pragmatic strategy to recover motion and pain relief.^10^, ^11^, ^12^ At the same time, concerns have been raised that aggressive mobilization and interval slides used in an attempt to achieve complete repair despite persistently elevated tension may compromise tendon-to-bone healing without clearly improving structural integrity.^12^^,^^18^^,^^19^ In our pooled analysis, structural failure rates did not differ significantly between techniques. However, interpretation should remain cautious because treatment allocation in the included studies was likely influenced by external factors such as repairability, tissue quality, and intraoperative decision-making.^2^^,^^9^^,^^10^^,^^15^^,^^17^^,^^18^^,^^21^ Finally, augmentation strategies including biceps-based procedures and patch/scaffold augmentation remain areas of ongoing investigation. Given the comparable PROMs observed between repair strategies in this review, the clinical and economic value of augmentation approaches requires further study before broader conclusions can be made.^4^^,^^14^^,^^18^^,^^20^^,^^22^

Structural endpoints warrant careful terminology. After a complete repair, post-operative imaging evidence of tendon discontinuity is reasonably interpreted as a true retear. After a partial repair, a residual defect is expected; therefore, post-operative imaging can only be interpreted as structural progression (enlargement of the residual defect or loss of repair continuity beyond the intended repair) when compared against the post-operative construct. Across included studies, post-operative imaging protocols and failure definitions varied, and some cohorts relied on symptom-based definitions, limiting cross-study comparability and strengthening the need for standardized structural reporting. Imaging modalities used for post-operative structural assessment also varied across studies and included magnetic resonance imaging‑based and ultrasound-based protocols, further contributing to heterogeneity in reported structural outcomes.

ROM outcomes should also be interpreted with an appreciation of measurement error and examiner variability. Even when a goniometer is used under standardized conditions, inter-rater standard error for common glenohumeral motions has been reported in the range of approximately 6°-9°, and minimal detectable change can exceed 10°. Accordingly, small pooled between-group ROM differences—especially when heterogeneity is high or measurement positions differ—may fall within measurement noise and should not be overinterpreted.^32^

This study has limitations that reflect the current evidence base for large-to-massive rotator cuff tears. First, all included studies were non-randomized, introducing substantial risk of selection bias and confounding by indication. The decision to perform partial vs. complete repair was unlikely to be random but rather influenced by intraoperative reparability, tissue quality, tendon mobility, degree of retraction, and fatty infiltration. Consequently, the partial and complete cohorts may represent fundamentally different patient populations despite similar nominal tear size classifications (Table II). Second, tear definitions and baseline reporting were inconsistent (eg, ≥ 3-cm vs. ≥ 5-cm thresholds, variable tendon-count criteria), and standardized pre-operative healing-risk stratification (eg, Rotator Cuff Healing Index) was generally absent. Moreover, fatty infiltration and tendon-quality subgroup analyses were inconsistently reported and therefore could not be pooled. Third, outcome ascertainment varied: ROM is examiner-dependent with non-trivial measurement error; strength testing protocols differed across studies; and structural endpoints were heterogeneous in imaging modality, timing, and failure definitions. Fourth, cost-effectiveness outcomes were not evaluated in the included studies and therefore could not be assessed in the present review. Finally, several pooled estimates exhibited substantial statistical heterogeneity (I^2^ > 50%) and should be considered exploratory rather than definitive. Despite these limitations, the present synthesis suggests broadly comparable patient-reported outcomes following partial and complete repair, with complete repair demonstrating a modest but consistent advantage in objective strength outcomes. These findings should be interpreted within the context of the heterogeneous and non-randomized evidence base from which they were derived.

Future high-level studies comparing non-augmented vs. augmented partial repairs are warranted to better define the role of these techniques in bridging the gap between functional demands and anatomic limitations. Additionally, more extensive multicenter clinical studies with standardized surgical protocols, post-operative rehabilitation, and follow-up times would be of great benefit.

## Conclusion

Both partial and complete arthroscopic repair of large-to-massive rotator cuff tears yield meaningful post-operative improvement, with broadly comparable pain relief, ROM, and PROM-defined daily function across most low-heterogeneity outcomes. Complete repair provides a modest but consistent advantage in objective strength recovery. Across studies that performed post-operative imaging, imaging-defined structural failure/progression rates were similar; however, imaging protocols and structural definitions varied, and for partial repairs, the clinically relevant structural endpoint is progression/enlargement of the residual defect rather than the presence of a defect itself. These findings should be interpreted cautiously given the retrospective, non-randomized nature of the available evidence and the substantial risk of selection bias related to reparability and tear characteristics.

## Disclaimers:

Funding: No funding was disclosed by the authors.

Conflicts of interest: The authors, their immediate families, and any research foundations with which they are affiliated have not received any financial payments or other benefits from any commercial entity related to the subject of this article.

## Availability of data and material

All data are available within the manuscript and its supplemental files.
